# The Safety Limits Of An Extended Fast: Lessons from a Non-Model Organism

**DOI:** 10.1038/srep39008

**Published:** 2016-12-19

**Authors:** Fabrice Bertile, Laetitia Fouillen, Thierry Wasselin, Pauline Maes, Yvon Le Maho, Alain Van Dorsselaer, Thierry Raclot

**Affiliations:** 1 CNRS, UMR7178, 67037 Strasbourg, France; 2 Université de Strasbourg, IPHC, Laboratoire de Spectrométrie de Masse Bio-Organique, 25 rue Becquerel, 67087 Strasbourg, France; 3 Université de Strasbourg, IPHC, Département Ecologie, Physiologie et Ethologie, 23 rue Becquerel, 67087 Strasbourg, France

## Abstract

While safety of fasting therapy is debated in humans, extended fasting occurs routinely and safely in wild animals. To do so, food deprived animals like breeding penguins anticipate the critical limit of fasting by resuming feeding. To date, however, no molecular indices of the physiological state that links spontaneous refeeding behaviour with fasting limits had been identified. Blood proteomics and physiological data reveal here that fasting-induced body protein depletion is not unsafe “per se”. Indeed, incubating penguins only abandon their chick/egg to refeed when this state is associated with metabolic defects in glucose homeostasis/fatty acid utilization, insulin production and action, and possible renal dysfunctions. Our data illustrate how the field investigation of “exotic” models can be a unique source of information, with possible biomedical interest.

Protein wasting has been associated with metabolic disorders and death in disease-related starvation^1^. However, biomedical interest on caloric restriction and intermittent fasting has grown tremendously, due to their beneficial effects on lifespan and their protective effects against obesity, diabetes, and cardiovascular disease^2^. This interest has been reinforced by the recent demonstration that the effects of chemotherapy are enhanced and its side-effects diminished in mice if they have previously fasted for 48–60 hours^2^. At this stage and because of their small size, increasing body protein breakdown is likely to occur in non-obese, fasting mice^3^. This situation thus strengthens the need to better characterize those physiological and molecular changes that occur during late fasting.

Protein wasting is the hallmark of long-term fasting in laboratory and wild animals^4^,^5^,^6^, and in extremely malnourished, dying patients, where it has been involved in the so-called king penguin syndrome^7^. The survival of long-term fasting animals as well as that of malnourished patients can be ensured even if refeeding is initiated after the onset of protein catabolism^5^,^7^. However, death in humans and animals has been reported to occur only when body protein loss reaches about 30–50%^6^,^8^, a range obviously too wide for a meaningful anticipation. There is then no clear link between fasting-induced increase in protein breakdown and health deterioration. Definitely adjourning the possible debate on the safety of fasting would require the ability to recognize and anticipate, beyond protein loss, the critical limit during extended fasting, so that the condition can still be successfully reverted by refeeding. The current study was specifically designed to fill this gap using wildlife as an open laboratory.

Evidence is growing that resolving important human health issues may not be achieved only through studies in the mainstream of murine models, but would rather require studies in appropriate wild animal models^9^. To tackle the question of the safety of the king penguin syndrome (i.e. the limits of prolonged fasting), wild animals such as penguins are an obvious model of choice. Indeed, they make safe use of their body reserves during the various phases of their life cycle (e.g. during breeding or migration), and the general rules governing their body fuel utilization during extended fasting are similar to those observed in rodents and humans.

When breeding on land or ice, a male penguin endures an extremely long fast to incubate the egg, waiting for the female to return from feeding at sea to relieve him. In the fasting male, most of the energy requirements are met through the mobilization and oxidation of lipid fuels^4^,^5^,^6^. The reduction in body protein utilization, i.e. the so-called protein sparing phase 2, is considered as a key adaptation to long-term fasting in humans, other mammals, and birds^4^,^5^,^6^,^8^. However, once ∼80% of initial fat stores have been exhausted in both rats^5^ and birds^4^, protein utilization increases during the so-called phase 3. Then, the contribution of lipids to energy expenditure decreases, while that of body proteins increases by up to 55%^4^,^5^,^6^.

To the best of our knowledge, no incubating penguins have ever been reported to have died of starvation. This is due to a refeeding signal that occurs during phase 3 of fasting and promotes nest abandonment and departure of incubating males, if the female has not returned^4^. This signal presumably is part of a general defence mechanism in animals, since an increased locomotor activity, attributed to an increased drive for refeeding^10^, has also been observed in laboratory rats during phase 3 of fasting^11^. Investigations in penguins and rats indicate that the refeeding signal, the nature of which remains to be fully elucidated, operates below a certain body mass threshold and involves peripheral hormones such as leptin^12^ and corticosterone^13^. However, molecular indices of the physiological state that link spontaneous refeeding behaviour are still lacking.

We monitored male Adélie penguins (*Pygoscelis adeliae*) during their incubation fast under natural conditions. To minimize disturbance, incubating male Adélie penguins were captured and sampled only twice. The first capture took place at the pre-laying stage, i.e. before incubation started. Birds were recaptured upon their departure from the colony to refeed at sea, which followed either relief by the partner or nest abandonment. For the study of metabolic processes that integrate information across cells and tissues, large screening strategies provide a successful alternative to assays of predefined targets^14^, thanks to progress in mass spectrometry-based proteomics^15^. In addition, proteomics is powerful enough to enable analysis of proteins from “exotic” organisms not or not well represented in protein databases^16^,^17^. Using a high-throughput proteomics analytical strategy, our objective was therefore to identify plasma molecular indices of the physiological state that link spontaneous refeeding behaviour with fasting limits in Adélie penguins living under natural conditions.

## Results and Discussion

**Figure 1:**
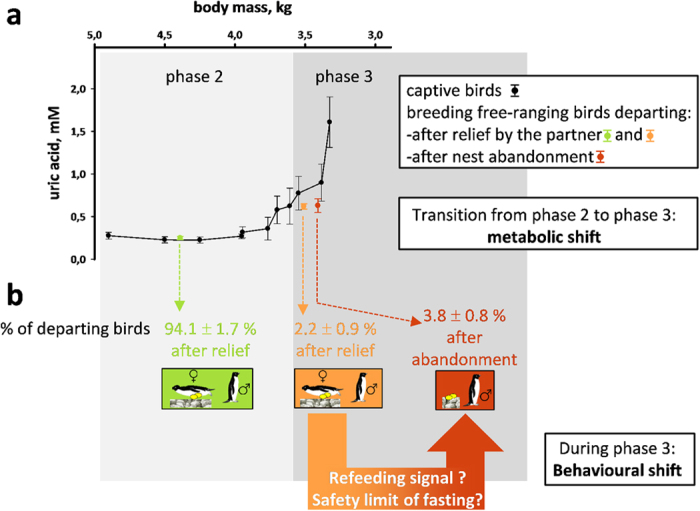
Uncoupling between the metabolic and behavioural shifts in fasting Adélie penguins. The transition from phase 2 to phase 3 of fasting is characterised by a metabolic shift reflected in changes in body mass and plasma metabolic profiles, including uric acid levels that reflect protein breakdown (**a**). Penguin body masses (variations not shown for better viewing) ranged from 4.9 ± 0.28 kg (beginning of phase 2) to 3.35 ± 0.11 kg (late phase 3). For captive animals, measurements were achieved from 6 birds monitored from phase 2 to the late phase 3 of fasting. For breeding wild birds (i.e. incubating males), % of departing birds were calculated over four austral summers (**b**), means ± SEM; n = 92–105/year). Departure to sea was either consecutive to relief by females or to nest abandonment. These data show that the spontaneous behavioural shift toward nest abandonment, which likely corresponds to the safety limit of fasting, occurs during phase 3 of fasting and is therefore not coupled with the metabolic shift occurring at the breakpoint from phase 2 to phase 3. Penguin drawings were made by Fabrice Bertile.

**Figure 2:**
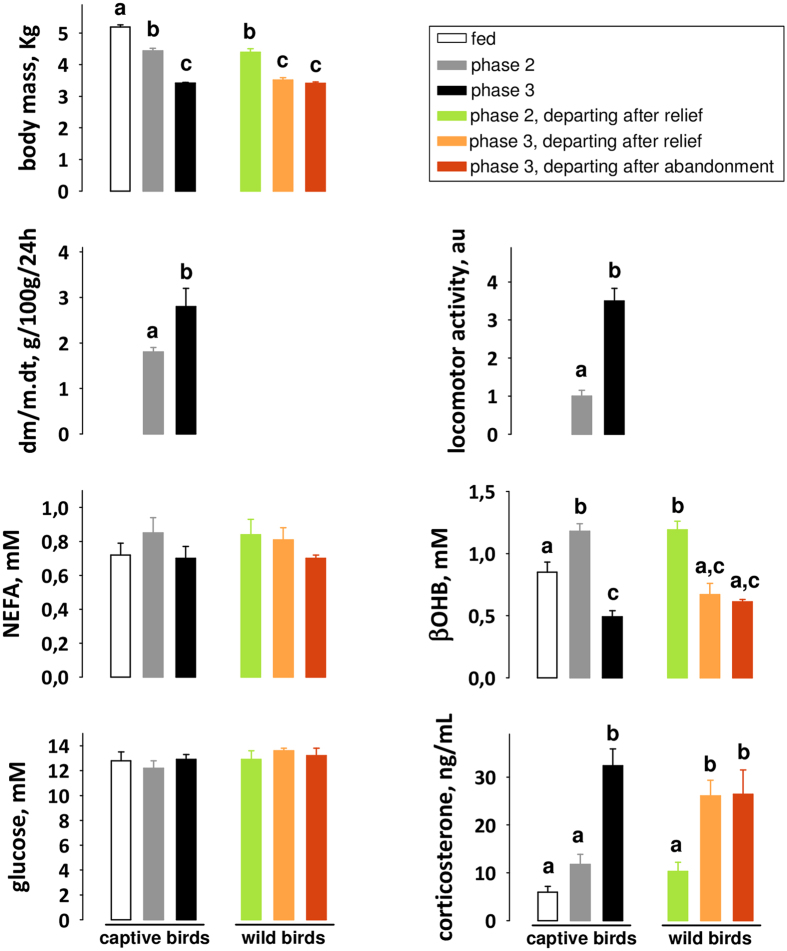
Metabolic profiles of male Adélie penguins in the fed and fasted states. The metabolic shift from phase 2 to phase 3 is characterized by plasma metabolite and hormone profiles, which drive changes in the respective contribution of fuels to energy needs. Results are means ± SEM (n = 6 per group, except for wild birds in phase 3 departing after relief where n = 3). To minimize disturbance of free-ranging birds and for relevance of the experimental protocol, only captive animals were equipped with a pedometer and weighed daily. Hence, Student’s t-test was used to compare the rate of daily body mass loss (dm/m.dt) and locomotor activity levels in captive birds (P < 0.05) and ANOVA followed by Tukey post-hoc tests were used for all other parameters in all birds (P < 0.05). Values that do not share the same letter are significantly different (P < 0.05). au: arbitrary unit. NEFA: non-esterified fatty acids; βOHB: β-hydroxybutyrate.

In order to accurately assess their metabolic status of wild penguins with respect to the three phases of fasting, we concurrently studied the precise time-course of fasting in captive birds, so that we obtained reference values, to which the samples from birds in the wild could be compared (Figs 1 and 2). Change from low to high rate of daily body mass loss, indicating increasing protein utilization as previously demonstrated for rats and birds^4^,^11^, allowed to characterize phase 2 and phase 3 of fasting in captive penguins (Fig. 2). High levels of lipid-derived fuels (i.e. non-esterified fatty acids and β-hydroxybutyrate) accompanied by low levels of protein degradation products (i.e. uric acid) in plasma samples confirmed phase 2 of fasting, while a rise in plasma corticosterone and uric acid levels confirmed phase 3 of fasting. In line with previous results^4^,^11^, our captive birds showed an increasing locomotor activity in phase 3 (3.5 times higher than during phase 2), reflecting the increased drive for refeeding. The comparison of the body mass and plasma profiles of penguins in the wild vs. captive birds showed that the vast majority of breeding birds (∼94%) were in phase 2 of fasting when they departed to refeed at sea, after being relieved by their mate (Fig. 1). Because their partner was delayed, the remaining ∼6% of breeding birds entered into the stage of increased protein breakdown (phase 3). Two-thirds of them abandoned incubation before being relieved by their partner (commonly reported situation). The other one third was relieved by their mate before departure (rarely reported situation). This indicates an uncoupling between the metabolic shift that occurs at the transition from phase 2 to phase 3 of fasting and the bird’s decision to leave for refeeding. The behavioural shift from incubation toward nest abandonment is thus not directly triggered by the metabolic shift, and its determinants have to be searched among molecular events that occur during the course of phase 3 of fasting (Figs 1 and 2).

**Figure 3:**
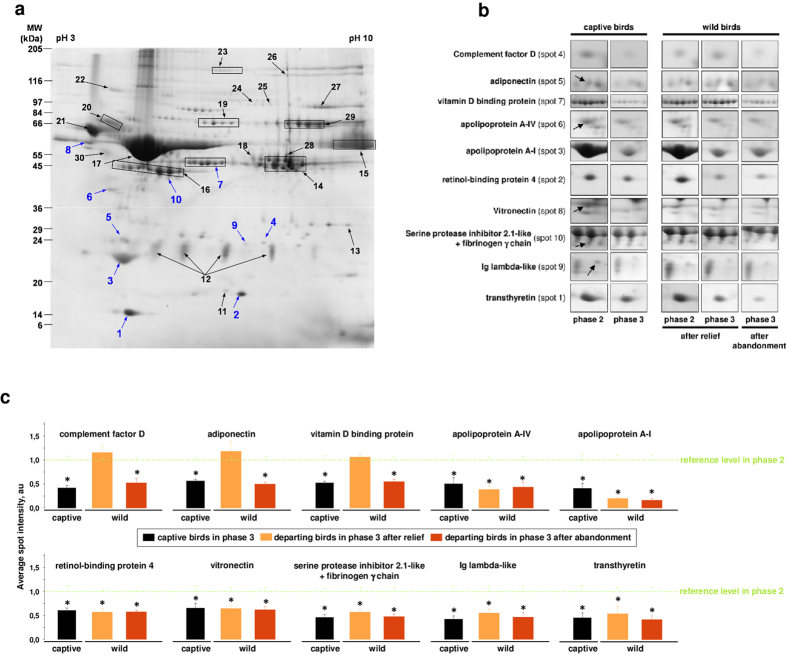
Differential plasma proteomics in wild animals characterizes the safety limit of an extended fast. A total of 36 unique avian proteins from 30 excised “individual or grouped (i.e. from isoforms; boxed areas) gel spots” were identified (**a**; see also Tables S1 and S2). The intensity of 10 protein spots (**a**, blue numbers), containing 11 distinct proteins, differed significantly between groups (**b**). The relative plasma abundance of these 10 proteins (**c**) is illustrated for phase 3 penguins with reference to corresponding phase 2 birds (green dashed line; the variation on reference levels in phase 2 penguins is given as green error bars). Data are means ± SEM (n = 6 per group, except for wild birds in phase 3 departing after relief where n = 3). Comparisons between captive 3 birds were performed using Student’s t-tests, while comparisons between breeding birds were performed using ANOVA followed by Tukey post-hoc tests. Significance (*) between groups was set at P < 0.05.

The proteomic comparison of plasma samples during phase 3 vs. phase 2 in captive birds revealed the downregulation of ten proteins during phase 3 (Fig. 3; Tables S1 and S2), of which five had not been reported in phase 3 fasting rats^18^. Among these ten downregulated proteins, most are known to be involved in the regulation of energy balance^19^; they also play a role in insulin resistance and lipid metabolism, immunity and inflammation, and bone metabolism^20^,^21^; they are furthermore linked with cardiovascular risks and haemostasis, liver failure, and renal pathologies^21^,^22^. Importantly, these phase 3 alterations in captive birds occurred at a fasting stage at which penguins can still be successfully refed^4^. Only seven of the ten proteins downregulated in captive birds were also downregulated (Fig. 3) in wild birds that entered phase 3 of fasting but left for refeeding after the return of their mate. These molecular blood changes likely are characteristic for the ultimate stage of fasting, albeit still compatible with refeeding. By contrast, the three remaining of the ten proteins were only downregulated in the wild phase 3 birds that were not relieved and, consequently, abandoned their nest. Changes in the expression of these 3 remaining proteins, namely vitamin D binding protein, complement factor D, and adiponectin (Fig. 3), should therefore reflect the point where the fast has to be interrupted before survival is jeopardized.

Since plasma complement factor D, also termed adipsin, is a serine protease that mediates acute inflammatory reaction^20^, its low levels would impair host defense during phase 3 of fasting, although increased susceptibility for infections in humans with a partial factor D deficiency is unlikely^23^. From studies in knockout mice, complement factor D deficiency has been linked to mesangial disease^22^ and β cell failure^24^. Thus, lowering levels of complement factor D during phase 3 could be a mechanism contributing to renal dysfunctions and/or reduced insulin secretion (i.e. impairment of glucose homeostasis) in late fasting.

Vitamin D binding protein protects animals from the numerous dysfunctions that may arise from the release of actin into the blood stream after cell lysis^21^,^25^. As fasting involves the wasting of most tissues, unusually high amounts of actin are without doubt released into the blood. The lower levels of vitamin D binding protein observed during phase 3 in abandoning vs. relieved birds suggests that the former are more sensitive to the deleterious effects of high actin levels. It is also probable that lower levels of vitamin D binding protein in the plasma reflect its reduced availability in the brain of abandoning phase 3 birds. Laboratory rodents with vitamin D deficiency exhibit alterations in their locomotor activity pattern, possibly due to changes in several neurochemical pathways^26^. Below a certain threshold, the circulating concentrations of vitamin D binding protein may therefore either contribute to or reflect impairments in vitamin D metabolism, which possibly contribute to increases in both locomotor activity and the drive for refeeding during phase 3.

The central administration of adiponectin decreases body weight by stimulating energy expenditure^27^. Adiponectin also peripherally enhances glucose utilization and fatty acid oxidation^19^, and initiates an insulin-sensitizing action^28^, while hypoadiponectinemia has been associated with accelerated muscle proteolysis^29^. Low adiponectin levels in abandoning penguins may therefore impair insulin sensitivity while allowing adaptive metabolic adjustments, such as energy sparing and tuning of fuel partitioning, to increase the safety margin associated with fasting duration. Low adiponectin may also promote survival by signalling deleterious nutritional and/or metabolic changes, e.g. to the brain, thus playing a pivotal role in the induction of refeeding behaviour during late fasting.

**Figure 4:**
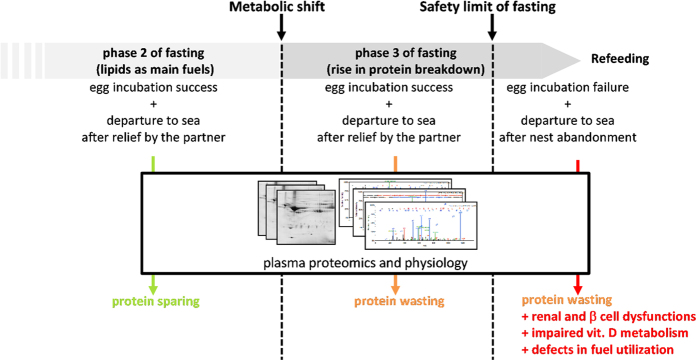
Protein wasting is not the only determinant of the safety limit of fasting. The low levels of adiponectin, complement factor D, and vitamin D binding protein only found in the plasma of phase 3 animals after nest abandonment show that fasting limits are achieved only when the rise in protein breakdown is associated with functional impairments and metabolic alterations (see text for details). The reasons for which only some penguins in phase 3 of fasting exhibit dysfunctions that, in addition to protein wasting, lead to nest abandonment remain to be determined.

Thus, when its partner is delayed, a penguin incubating its egg can safely extend its fast despite increasing protein wasting. This indicates that fasting-induced body protein depletion is not unsafe “*per se*”, and is not the sole determinant of the safety limit of fasting. Our data show that a fasting penguin only abandons to refeed when energy deprivation develops into a critical metabolic situation with the occurrence of functional impairments, which therefore characterize the safety limits of an extended fast (Fig. 4). Occurrence of defects in glucose homeostasis/fatty acid utilization and insulin action, an imbalance in vitamin D metabolism, and possible renal and β cell dysfunctions can be depicted by specific changes in circulating adiponectin, vitamin D binding protein, and complement factor D. Identification of similar biomarkers associated with catabolic pathophysiological states in humans might favour anticipation and/or management of protein wasting disorders. By feeding the understanding of the physiological safety limits of prolonged fasting, the data we report here extend beyond the use of exotic animals in the laboratory as a new source of biomedical information^9^. Indeed, they would not have been obtained in captivity as they relate to behavioral changes spontaneously occurring in wild animals under natural conditions. The analysis of proteins from “exotic” organisms could also be validly used in future studies examining whether and to what extent environmental factors such as urbanization, pollution or malnutrition can affect the physiological or the stress responses of key species submitted to these constraints. In this framework, the screening of molecular changes may also serve as biomarkers for endogenous responses to environmental conditions.

## Methods

### Study Protocol

Breeding Adélie penguins (*Pygoscelis adeliae* ; 92–105 pairs/year) were monitored during four austral summers (2004–2008) in Dumont d’Urville Station (66°40′S, 140°01E), Adélie land, Antarctica. All methods were performed in accordance with the relevant guidelines and regulations, being approved by the independent ethics committee evaluating Antarctic projects (Comité d’Ethique Midi-Pyrénées) and the Interdepartmental Committee of polar environment. The study was authorised (decrees 2004-195, 2005-203, 2006-73, and 2007-157) by the Prefect of Terres Australes et Antarctiques Françaises. The mass spectrometry proteomics data have been deposited to the ProteomeXchange Consortium via the PRIDE^30^ partner repository with the dataset identifier PXD004808.

At the pre-laying stage, both members of each pair were captured, weighed and individually identified. Penguins were sexed by cloacal inspection before egg laying and also by observation of incubation behaviour. Intense observation (2–4 hours intervals) of the nests provided a record of incubation routine and laying date. Males were recaptured at the end of the first incubation shift after their partner had returned or when they abandoned the nest. Body weight was recorded, blood was collected using EDTA tubes, and plasma was prepared and kept frozen at −80 °C. Plasma samples to be used for further proteomics analysis were stored and shipped to the lab in liquid nitrogen.

In the current study, some male Adélie penguins started a reproductive cycle but abandoned prematurely due to extrinsic factors not linked to body condition, such as flooding or predation. Eighteen of these failed breeders were captured, randomly divided into three different groups (n = 6 each), kept in a pen (5.2 m * 2.4 m) and equipped with pedometers (Dista 300, Decathlon, France) for the daily recording of locomotor activity during fasting. Body weight was also recorded daily. The identification of phase 2 and phase 3 of fasting was based on the kinetics of body mass loss and locomotor activity^4^,^10^,^11^. Blood was sampled in phase 2 and in phase 3 animals (n = 6/group), and then treated as described above.

### Experimental groups

The metabolic status of all birds was systematically validated by plasma metabolite and hormone assays (see below). It was not possible to adjust precisely the sample size and ensure adequate statistical power, because phase 3 birds are rare and can be missed when they depart to sea. Sampling over four austral summers was necessary to obtain few or enough wild breeding phase 3 birds. The number of penguins for which we further analysed the plasma proteome was as follows: n = 6 for relieved birds in phase 2, n = 3 for relieved birds in phase 3 and n = 6 for abandoning birds in phase 3. Additionally, captive fed, phase 2 and phase 3 birds (see above; n = 6/group) were also sampled for further analysis.

### Plasma metabolites and hormone assays

The metabolic status of each animal was assessed through assays of circulating metabolite and hormone levels. Enzymatic measurements were carried out using kits from Sigma Diagnostics (St. Louis, MO, USA; non-esterified fatty acids, glucose and uric acid) and Randox Laboratories (Crumlin, UK; β-hydroxybutyrate). Corticosterone concentrations were determined by ELISA (AssayPro, St. Charles, MO, USA).

### Plasma proteomics

Circulating albumin was eliminated using the vivapure anti-HSA kit from Vivascience (Palaiseau, France). Plasma proteins were separated by two-dimensional electrophoresis, as previously reported^18^, except that the second dimension was performed on 7–18% acrylamide gels. All samples were run in duplicate. Protein spots visualization, 2-D gel image acquisition and analysis were performed as previously described^18^, using PDQUEST™ software (version 8.0; Biorad, Hercules, CA, USA). After excision of gel slices, in-gel reduction, alkylation, and trypsin digestion, the extracted peptides were analyzed by a nanoLC-Chip/MS/MS approach, using an Agilent 1100 series HPLC-Chip/MS system (Agilent Technologies, Palo Alto, USA) coupled to an HCT™ Ultra ion trap (Bruker Daltonics Gmbh, Bremen, Germany).

The chromatographic separations were conducted on a HPLC chip composed of an enrichment column (Zorbax 300SB-C18, 40 nL, 5 μm) and a reverse-phase separation column (Zorbax 300SB-C18, 43 mm × 75 μm, 5 μm). The flow rate was set to 3.75 μL/min for loading of the samples with acetonitrile 2%/HCOOH 0.1% in water. Mobile phase solvents were acetonitrile 2%/HCOOH 0.1% in water (A) and water 2%/HCOOH 0.1% in acetonitrile (B). A linear gradient from 8 to 40% B in 7 min. was used to achieve the chromatographic separation, with a flow rate set to 300 nL/min.

The mass spectrometer was operated in positive mode and the voltage applied to the capillary cap was optimized to −1750 V. Scan rate was 8100 m/z and 26000 m/z per sec. for MS and MS/MS scanning, respectively. A total of 4 and 6 scans were averaged to obtain a MS and MS/MS spectrum, respectively. Through an automatic switching between MS and MS/MS modes, the three most abundant doubly charged ions were selected on each MS spectrum for further isolation and fragmentation using CID. The complete system was fully controlled by the ChemStation Rev B.01.03 (Agilent Technologies) and EsquireControl 6.1 (Bruker Daltonics Gmbh) softwares.

MS/MS data were interpreted using a local Mascot™ server (version 2.5.1, Matrix Science, London, UK). Spectra were searched with a tolerance of 0.5 Da on mass measurements, both in MS and MS/MS modes. One trypsin missed cleavage per peptide was allowed, and carbamidomethylation of cysteine, oxidation of methionine, and acetylation of protein N-termini were specified as variable modifications. Spectra were searched against a target-decoy version (July 2015) of the NCBInr database restricted to *Aves* (Taxonomy ID: 8782; 3270982 target+decoy entries). Using Scaffold software v.3.6.5 (Proteome software Inc., Portland, OR, USA), stringent filtering criteria were established to identify proteins and estimate a false discovery rate of identification (target-decoy strategy). Hence, protein identification was considered for MS/MS ion scores higher than 39.7 when based on ≥ 2 peptides, while a MS/MS ion score higher than 57 was required for single peptide-based identifications.

Complete or partial amino acid sequences were determined on the non-assigned MS/MS spectra through automatic *de novo* sequencing, using the PEAKS Studio software (v4.5; Bioinformatics Solutions, Waterloo, Canada). All deduced sequence tags were submitted to the MS-BLAST programme to perform cross-species protein identifications on the basis of sequence similarity.

### Statistical analysis

The normal distribution of the measured variables was tested using the Shapiro-Wilk test (P > 0.01), and homoscedasticity using the Bartlett test (P > 0.01). Reproducibility between duplicate 2-D gels was tested using Student’s t-test applied to the intensities of each spot (P < 0.05). Spot volume intensities obtained for captive and wild penguins in phase 3 were normalized to the values measured for captive birds in phase 2 and wild birds relieved in phase 2, respectively. Densitometric data were normalized to phase 2 birds, which were assigned an arbitrary value of one. Values are presented as means ± SEM (n = 3–6/group). The R software environment v2.8.1 (http://cran.r-project.org/) was used for statistical comparisons between groups. Depending on the number of groups considered, we used either Student’s t-test or ANOVA followed by Tukey post-hoc tests. The level of significance was set at p < 0.05.

## Additional Information

**How to cite this article**: Bertile, F. *et al*. The Safety Limits Of An Extended Fast: Lessons from a Non-Model Organism. *Sci. Rep.* **6**, 39008; doi: 10.1038/srep39008 (2016).

**Publisher's note:** Springer Nature remains neutral with regard to jurisdictional claims in published maps and institutional affiliations.

## Supplementary Material

Supplementary Tables
