# Supplementary material for: The Safety Limits Of An Extended Fast: Lessons from a Non-Model Organism
Source: Sci Rep. 2016 Dec 19;6:39008. doi: 10.1038/srep39008 (PMC5171797; doi:10.1038/srep39008)
Supplement: Supplementary Tables [file srep39008-s1.pdf]

The Safety Limits Of An Extended Fast: Lessons from a Non-Model Organism

Fabrice Bertile[1,2*], Laetitia Fouillen[1,2]†, Thierry Wasselin[1,2], Pauline Maes[1,2], Yvon Le Maho[1,3], Alain Van Dorsselaer[1,2] and Thierry Raclot[1,3]

[1] CNRS, UMR7178, 67037 Strasbourg, France.

[2] Université de Strasbourg, IPHC, Laboratoire de Spectrométrie de Masse Bio-Organique, 25 rue Becquerel, 67087 Strasbourg, France.

[3] Université de Strasbourg, IPHC, Département Ecologie, Physiologie et Ethologie, 23 rue Becquerel, 67087 Strasbourg, France.

*Correspondence to: Email: fbertile@unistra.fr (F.B.).

† Current address: CNRS, Université de Bordeaux, UMR5200, Laboratoire de Biogenèse Membranaire, 71 Avenue Edouard Bourlaux, 33140 Villenave d'Ornon, France.

Supplementary Information

Table S1. List and annotations of the plasma proteins identified on 2-D gels in male Adélie penguins

Table S2. Annotations relative to single peptide-based protein identifications, with corresponding annotated mass spectra

Table S1. List and annotations of the plasma proteins identified on 2-D gels in male Adélie penguins

Spot N°	Protein	NCBI Accession.version	Taxonomy	pI / MW (Da)	Number of peptides & tags / seq coverage (%)	Sequences of all identified peptides	Mascot MS/MS ion score	MS-Blast HSP score
1	Transthyretin	KFW72348.1	Pygoscelis adeliae	5.07 / 16,391.42	13 / 76.7	AADGSWQDFATGK / VEFDTSSYWK / GLGLSPFHEYADVVFTANDSGHR / HYTIAALLSPFSYSTTAVVSDPQE / GSPAANVAVK / KAADGSWQDFATGK / TTEYGEIHELTTEEKFVEGIYR	110 / 76.1 / 84.9 / 113 / 56.6 / 89 / 87.9	
						APLVSHGSVDSK / GSPAANVAVK / KAADGSWQDFATGK / TAVVSDPE / VEGLYR / VFTANDSGHR		81 / 67 / 103 / 50 / 44 / 74
	Chain A, Transthyretin	1TFP_A	Gallus gallus	5.08 / 14,160.3	5 / 60.8	APLVSHGSVDSK / GLGLSPFHEYADVVFTANDSGHR / HYTIAALLSPFSYSTTAVVSDPQE / VEFDTSSYWK / GSPAANVAVK	60.9 / 55.4 / 112 / 63 / 56.6	
	Transthyretin	P27731.1	Gallus gallus	5.07 / 16,309.36	6 / 40.0	APLVSHGSVDSK / GSPAANVAVK / KAADGSWQDFATGK / TAVVSDPE / VEGLYR / VFTANDSGHR		81 / 67 / 97 / 50 / 39 / 74
	PREDICTED: transthyretin	XP_010005130.1	Chaetura pelagica	5.23 / 16,425.5	11 / 65.3	AADGSWQDFATGK / GLGLSPFHEYADVVFTANDSGHR / HYTIAALLSPFSYSTTAVVSDPKE / IEFDTSSYWK / KAADGSWQDFATGK	104 / 84.9 / 113 / 62.1 / 78.5	
						APLVSHGSVDSK / GSPAANVAVK / KAADGSWQDFATGK / TAVVSDPE / VEGLYR / VFTANDSGHR		81 / 67 / 103 / 49 / 39 / 74
2	PREDICTED: retinol-binding protein 4	XP_009333082.1	Pygoscelis adeliae	6.08 / 22,778.71	6 / 56.3	KDPEGLFLQDNVVAQFTVDENGQMSATAK / LFNNWDVCADMIGSFTDTEDPAK / QLNEDGTCADSYSFVFSR / GNDDHWVVDTDYDTYALHYSCR / YSGTWYAMAK / YWGVASFLQK	104 / 133 / 125 / 60.4 / 57.5 / 54.5	
	Chain A, Chicken Plasma Retinol-binding Protein	1IIU_A	Gallus gallus	5.62 / 20,079.52	6 / 64.4	ELNEDGTCADSYSFVFSR / KDPEGLFLQDNVVAQFTVDENGQMSATAK / LFNNWDVCADMIGSFTDTEDPAK / GNDDHWVVDTDYDTYALHYSCR / YSGTWYAMAK / YWGVASFLQK	94 / 104 / 133 / 71.1 / 57.5 / 54.5	
3	PREDICTED: apolipoprotein A-I	XP_010722021.1	Meleagris gallopavo	5.58 / 30,612.11	16 / 42.8	DAIAQFESSAVGK / DMVDVYLETVK / VVEQLSNLR / GIPQAAEYQAK / IRDMVDVYLETVK	87.5 / 75.7 / 72.2 / 68.9 / 68.6	

						LADNLDTLSAAAAK / LTPVAEEAR / GIPQAAEYQAK/ VVEQLSNLR / WTEELEQFR / EDPQAPLER / EDAMPYYK / YYQEVR / PLVQQYVER / YKQRLLP / TPLVKDF		81 / 63 / 81 / 62 / 67 / 48 / 41 / 40 / 39 / 34 / 35
	PREDICTED: apolipoprotein A-I-like	XP_009894232.1	Charadrius vociferus	8.50 / 29,566.88	14 / 39.6	DAIAQFESSAVGK / FITLLDDLQK / GIPQAAEYQAK / VVEQLSNLR	87.5 / 62.5 / 68.9 / 72.2	
						LADNLDTLSAAAAK / LTPVAEEAR / GIPQAAEYQAK/ VVEQLSNLR / WTEELEQFR / EDAMPYYK / YYQEVR / PLVQQYVER / YKQRLLP / TPLVKDF		81 / 63 / 81 / 62 / 67 / 41 / 40 / 39 / 34 / 35
	PREDICTED: apolipoprotein A-I	XP_009272065.1	Aptenodytes forsteri	8.38 / 27,219.13	13 / 37.9	LADNLDTLSAAAAK / VVEQLSNLR / GIPQAAEYQAK	122 / 72.2 / 68.9	
						LADNLDTLSAAAAK / GIPQAAEYQAK/ WTEELEQFR/ VVEQLSNLR / LTPVAEEAR / LAPVAKDLK / EDAMPYYK / TPLVKDF YYQEVR / PLVQQYVER / YKQRLLP		89 / 81 / 67 / 62 / 55 / 43 / 41 / 40 / 35 / 33
4	PREDICTED: complement factor D	XP_009467440.1	Nipponia nippon	6.55 / 24,580.88	2 / 14.8	DDLLLLQLEEK / GDSGGPLVCSGVAEGVVTAGSR	99.2 / 96.5	
5	PREDICTED: adiponectin	XP_009333104.1	Pygoscelis adeliae	5.23 / 26,650.74	10 / 22.9	APHPNVPIR / AVVFTYDQFQK / IFYNEQSHYDASTGK / SAFSVGLTER	56.2 / 62.8 / 84.4 / 66.6	
						SAFSVGLTER / APHPNVPLR / EQSHYDA / GAFVYR / VFTFMQFQ / GHNGL		68 / 67 / 55 / 46 / 39 / 34
6	PREDICTED: LOW QUALITY PROTEIN: apolipoprotein A-IV	XP_009271768.1	Aptenodytes forsteri	4.74 / 37,955.46	9 / 29.5	AQVGQSVEELR / ETVDQLQQAEITK / LAASSEELR / LQDNAESIQASLSPYADR / MSPYAEELR / QLESLTAQMER / QLNTLLETK	97.4 / 86.3 / 66.1 / 78.2 / 56.5 / 71.5 / / 55.7	
						FAQEVKDGLNR / YFTELGSNAK		63 / 72
	PREDICTED: apolipoprotein A-IV	XP_011600120.1	Aquila chrysaetos canadensis	4.94 / 40,901.94	9 / 24.8	AQLSPLAQELR / AQVGQSVEELR / ETVDQLQQAEITK / LAASSEELR / MSPYAEELR / QLESLTAQMER / LAPLAQQLDER	66.3 / 97.4 / 86.3 / 66.1 / 56.5 / 71.5 / 79.2	
						QEAAPL / YFTELGSNAK		29 / 72
7	PREDICTED: Vitamin D binding protein	XP_009322325.1	Pygoscelis adeliae	Sequence contains several consecutive undefined AA. Its pI and Mw cannot be computed	35 / nd	DKVCQEFQALGK / HTTVPDAFLGK / TLSLLTLLSNR / VCQEFQALGK / ACGALLAR / DASACLDSK / MPGASFEDLFPLAEDAAEVFSQCCDSVAED CMQK / FLHEYASSYGQAPLPVLLGSTR / TFLSMVSTCCISPAPTSCFLK / YLQPSNEELCQAFK / TLTIIANSR	82.8 / 68.1 / 75.9 / 75.3 / 63.3 / 58.2 / 57.8 / 56.9 / 78.6 / 94.8 / 65.5	
						GCCAQEGLEKK / APLPVLLGSTR /		71 / 71 / 87 /

						CLSPAPTSCFLK / CSNGSPFPAHPGT / DFFDRFL / ECCSAK / EPSNEELCKAFK / FADCCSGK / FNQCCSDVAEDC / FSYLTSLAQK / GEAPLPVLL / HPPQQLP / HTTVPDAFLGK / LPEAQQPTNEQLC / MESTCC / RFVHE / RYLFELAR / SACLD / TLLANSR / TLSLLTLLSNR / VAEDCFQ / VCQEFQALGK / YDASE / YLKPSNE		33 / 39 / 47 / 73 / 62 / 68 / 61 / 51 / 54 / 79 / 86 / 38 / 34 / 59 / 35 / 45 / 67 / 43 / 74 / 38 / 42
		XP_009287230.1	Aptenodytes forsteri	8.02 / 54,878.22	45 / 63.9	LPEAQKPTNEQLCGEEGAR / TLTIIANSR / VCQEFQALGK / TLSLLTLLSNR / HPPQQLPR / DKVCQEFQALGK / YLQPSNEELCQAFK / HTTVPDAFLGK / ESLMQTMPKASPELLGQLVEQR / MPGASFEDLFPLAEDAAEVFSQCCDSVAEDCMQK / FLHEYASSYGQAPLPVLLGSTR / TFLSMVSTCCISPAPTSCFLK / ASPELLGQLVEQR	95.5 / 65.5 / 75.3 / 88.1 / 61 / 82.8 / 94.8 / 72.1 / 52.6 / 57.8 / 64 / 78.6 / 56.6	
						AGCCAQEGLEKK / APLPVLLGSTR / CLSPAPTSCFLK / CSNGSPFPAHPGT / DFFDRFL / ECCSAK / EPSNEELCKAFK / FADCCSGK / FASTCCPPNS / FNQCCSDVAEDC / FSYLTSLAQK / GEAPLPVLL / HPGTA / HPPQQLP / HTTVPDAFLGK / LCGQYTALNFLDFK / LCLAAL / LPEAQQPTNEQLC / LPPFLEK / MESTCC / PCCEDAGSSALSAK / ETMPEASPELLANLVEQR / RFVHE / RYLFELAR / SACLD / STCCPPNSPPLYC / TLLANSR / TLSLLTLLSNR / VAEDCFQ / VCQEFQALGK / YDASE / YLKPSNE		77 / 71 / 87 / 77 / 39 / 47 / 73 / 56 / 78 / 68 / 61 / 51 / 36 / 54 / 79 / 90 / 37 / 86 / 38 / 38 / 69 / 92 / 34 / 59 / 35 / 104 / 45 / 67 / 43 / 74 / 38 / 42
	PREDICTED: Vitamin D binding protein, partial	XP_010159925.1	Eurypyga helias	7.42 / 50,843.34	34 / Because sequence is “partial”. its coverage was not computed	QQMGEELPPFLEK / TLTIIANSR / VTFSYLTSLAQK / VSSELGEACDGGSCLLG / HPPQQLPR	64.5 / 63.4 / 87.6 / 107 / 61	
						AGCCAQEGLEKK / APLPVLLGSTR / CLSPAPTSCFLK / CSNGSPFPAHPGT / DFFDRFL / ECCSAK / EPSNEELCKAFK / FADCCSGK / FASTCCPPNS / FNQCCSDVAEDC / FSYLTSLAQK / GEAPLPVLL / HPGTA / HPPQQLP / HTTVPDAFLGK / LCGQYTALNFLDFK / LCLAAL / LPEAQQPTNEQLC / LPPFLEK / MESTCC / PCCEDAGSSALSAK / ETMPEASPEL / RYLFELAR / STCCPPNSPPLYC / TLLANSR /		63 / 71 / 70 / 74 / / 39 / 41 / 72 / 52 / 68 / 68 / 69 / 51 / 36 / 54 / 64 / 95 / 37 / 76 / 50 / 38 / 69 / 57 / 59 / 94 / 45 / 62 / 43 / 53 / 42

						TLSLLTLLSNR / VAEDCFQ / VCQEFQALGK / YLKPSNE		
	PREDICTED: LOW QUALITY PROTEIN: vitamin D-binding protein	XP_009873081.1	Apaloderma vittatum	8.15 / 52,737.52	34 / 57.2	ADFASTCCPPNSPPLYCASK / FLHEYASSYGQAPLPVLLGSTR / HPPQQLPR	79.2 / 64 / 61	
						AGCCAQEGLEKK / APLPVLLGSTR / CLSPAPTSCFLK / CSNGSPFPAHPGT / DFFDRFL / ECCSAK / EPSNEELCKAFK / FADCCSGK / FASTCCPPNS / FNQCCSDVAEDC / FSYLTSLAQK / GEAPLPVLL / HPGTA / HPPQQLP / HTTVPDAFLGK / CGQYTALNFLDFK / LCLAAL / LPEAQQPTNEQLC / LPPFLEK / MESTCC / PCCEDAGSSALSAK / PELLGKLVEQR / RFVHE / RYLFELAR / STCCPPNSPPLYC / TCCAD / TLLANSR / TLSLLTLLSNR / VAEDCFQ / VCQEFQALGK / YLKPSNE		61 / 71 / 68 / 79 / / 39 / 47 / 73 / 46 / 78 / 68 / 69 / 51 / 36 / 54 / 64 / 75 / 37 / 68 / 39 / 38 / 69 / 56 / 46 / 34 / 59 / 104 / 41 / 36 / 57 / 43 / 53 / 37
8	PREDICTED: Vitronectin	XP_009463431.1	Nipponia nippon	5.18 / 50,508.40	7 / 18.2	CQCDTLCVYYQSCCSDYSTVCK / FDDGALDPGYPR / LISDVWGIEGPIDAAFTR / YFYELDETSVRPGYPK	66.9 / 87.5 / 87.8 / 70.4	
						HEPTQ / EPEELCSR / DYSTVCK		33 / 49 / 55
	PREDICTED: LOW QUALITY PROTEIN Vitronectin	XP_010283829.1	Phaethon lepturus	5.74 / 50,784.53	7 / 12.2	FDDGALDPGYPR / LDAAMAGR / LISDVWGIEGPIDAAFTR / YFYELDETSVRPGYPK	87.5 / 55.6 / 87.8 / 70.4	
						HEPTQ / EPEELCSR / DYSTVCK		33 / 61 / 46
9	PREDICTED: LOW QUALITY PROTEIN: immunoglobulin lambda-like polypeptide 1	XP_009329120.1	Pygoscelis adeliae	6.41 / 24,855.74	1 / 8.5	QSNNQYMASSYLTLSASDWK	97.6	
10	PREDICTED: serine protease inhibitor 2.1-like	XP_009330653.1	Pygoscelis adeliae	5.92 / 47,286.14	4 / 12.4	LGFDGLTETR / ALLEIDEAGTEAAGATAIILTK / ATSTTLFMGK / STTLSQIFER	82.4 / 127 / 58.1 / 65.9	
	PREDICTED: fibrinogen gamma chain	XP_009328073.1	Pygoscelis adeliae	5.44 / 47,138.43	5 / 27.8	DNCCILDER / DTAEIQETTGR / KGTADYAVFK / LDGSEDFR / EGFGHLSPDDTTEFWLGNEK	61.5 / 70.5 / 67.3 / 59 / 61	
11	PREDICTED: lipocalin-like	XP_009288939.1	Aptenodytes forsteri	9.23 / 24,741.85	9 / 31.5	EQGLTDEEILILPR / MCTTVISATADGNLEVTSTYPK / STGSSTMVLLYSR	97 / 145 / 81.2	

						LSATADGNLEVTGMYPK / GSSTMVLLYSR / ALVATQLSK / SYTSPR / ELLLLP / LPVQTD		98 / 77 / 57 / 45 / 36 / 38
12	PREDICTED: LOW QUALITY PROTEIN: immunoglobulin lambda-like polypeptide 1	XP_009329120.1	Pygoscelis adeliae	6.41 / 24,855.74	1 / 8.5	QSNNQYMASSYLTLSASDWK	108	
	Ig lambda chain V-1 region, partial	KFW61302.1	Pygoscelis adeliae	6.19 / 6,395.03	1 / Because sequence is “partial”. its coverage was not computed	VPGSAPVTVIYANDK	114	
	Ig lambda-1 chain C regions, partial	KFQ94409.1	Nipponia nippon	6.91 / 11,720.02	1/ Because sequence is “partial”. its coverage was not computed	ATLVCLLGDFYPR	97.5	
13	lactate dehydrogenase A	XP_011574069.1	Columba livia	8.19 / 36,602.51	1 / 3.6	VIGSGCNLDSAR	68.7	
14	PREDICTED: Fibrinogen beta chain	XP_009328071.1	Pygoscelis adeliae	8.11 / 54,568.40	18 / 52.7	AVVDSLH / DNDGWLTADSR / LENAIATQVDYCR / LSETSTTFYEYMTVLDNK / SPCVVSCNIPVVSGK / IGPTEVLIEMEDWNGDK / NYCDTPGEYWLGNDK / YQLSVSNYK / AYCDMQTDNGGWTLIQNR / DNLDNNIPSSLR / HGTDDGVVWMNWK / HPLDELGVLCPTGCELQTTLVK / TMTIHNGMFFSTYDR / VSAHYGGFTIQNEGNK / YYWGGTYSWDMAK / KDNDDILSQYNTEVELHYNYIK / EDGGGWWYNR / GFGNVAK	53.3 / 88.9 / 85.1 / 124 / 91.7 / 115 / 94.6 / 71.2 / 91.2 / 58.1 / 103 / 72.4 / 58.7 / 97.6 / 82.8 / 53.5 / 61.7 / 39.7	
15	Ig gamma chain C region, partial	EMC81142.1	Columbia livia	4.72 / 10,826.22	1 / Because sequence is “partial”. its coverage was not computed	GFYPEDVSVEWQK	76.7	
	Ig heavy chain V-III region	KFQ92087.1	Nipponia Nippon	5.10 / 6,711.43	1/ Because sequence is	STVTLQMNSLK	59.5	

	VH26, partial				“partial”. its coverage was not computed			
		KFP43328.1	Chlamydotis macqueenii	8.11 / 6,122.75	1 / Because sequence is “partial”. its coverage was not computed	ADDTATYFCAK	72.4	
16	PREDICTED: fibrinogen gamma chain	XP_009328073.1	Pygoscelis adeliae	5.44 / 47,138.43	26 / 73.8	DNCCILDER / IIEEIIR / FGSYCPTTCGIADFFNK / GTADYAVFK / QTLPNSVDDFTKR / DTAEIQETTGR / FAGNCAEQDGSGWWMNR / IAQLESHCQEPCR / DASPAGYDNGIIWATWR / IELEDWSGK / KGTADYAVFK / LDGSEDFR / LPVDGQQHNLGSAK / YHLTMDNELQEMER / EGFGHLSPDDTTEFWLGNEK / LTYAYFIGGEAGDAFGGFDFGDDPSDK / QTLPNSVDDFTK / VGSEEDKYR / YYIGGVYTSK / QSFLVYCEIDSYGNGWTVIQR / YENTILSHESTIQQLTDTHMLNSK / KIIEEIIR / IHSITIQSTLPYTLR / SFTYHNGMQFSTYDNDNDK	78.1 / 58 / 92.7 / 70.9 / 70 / 89.4 / 106 / 76.6 / 113 / 65.6 / 73.6 / 64.6 / 98.8 / 103 / 88.5 / 130 / 74.8 / 82.6 / 71.2 / 133 / 59.1 / 57.8 / 104 / 56.3	
						VYCELDSYGNGWTVLKR / TLPYMLR /		121 / 39
	PREDICTED: alpha-1-antitrypsin-like	XP_009330651.1	Pygoscelis adeliae	5.41 / 48,275.19	17 / 52.2	DSDFLLDAK / ETVSNWMESLEK / GNAAALFVLPDEGTMK / LDSDTVMVLVNYIFFK / DENLSCWVVEIPYK / FLDDVTNFYNSEAISSNFQNSPEATK / MGVTEVFSDEADLSGVAEK / FSISGSYDVK / IAPSNANFAFR / IVNPAAKED / TTYGILFMGK / IVNPAAK / KLDSDTVMVLVNYIFFK / SNLNDEQQEQK/ EVQLSIGNALFIDDR / GYWEKPFNNLVTR / KIYLDLPK	69.8 / 88.3 / 84.8 / 114 / 88.2 / 80.6 / 144 / 75.8 / 94.3 / 69.5 / 70.3 / 40.7 / 85.6 / 62 / 92.6 / 58.7 / 57.7	
	PREDICTED: serine protease inhibitor 2.1-like	XP_009330653.1	Pygoscelis adeliae	5.92 / 47,286.14	4 / 15	LGFDGLTETR / NVFFSPMSISTAFAMLAVGAK / ALLEIDEAGTEAAGATAIILTK / ATSTTLFMGK	93.7 / 137 / 125 / 58.8/	
	PREDICTED:alpha-1-antiproteinase	XP_009330650.1	Pygoscelis adeliae	5.94 / 47,437.45	6 / 15.7	GEAIACLK / QLEQTLVK / DMDPQTVMLLASFVFFK / LVPNNADFAFQFFK / EFFVDAETTVK /	54.8 / 62.7 / 122 / 83.9 / 54.8 / 59	

	2-like					QINDYIER		
17	PREDICTED: serum albumin isoform X2	XP_009323154.1	Pygoscelis adeliae	5.53 / 68,668.91	31 / 54.7	ESDVGACLDEK / TIADGFTAMVDK / AELMTYMCSK / DSYGAMADCCSK / LPCSEGYLSIVMQDMcR / MPQVSTDTLIEIGK / QSDIDTCFGEEGANLIVQSR / SQCIIEADFDDQPEGLPSLVER / YNDLKEETFK / RHPEFSTQLILR / RPCFTAMGVDTK / ECCEGDMVECMDDR / RQETTPINDNVSHCCSDSYAYR / TNCELLTAQGETGFLK / AVTMITFAQYLQR / KMPQVSTDTLIEIGK / APLSEMLK / LLVNLIK / YVPPAFDPEMFSFDEK / LVKDVVDLAQK / MPQVSTDTLIEIGKK / ADKLALLSQK / CVANEDAPECTKPLPSIFLDEICQVEK/ REPFLYAPTILSLAADYEHALQTCCK / RLPCSEGYLSIVMQDMCR / QETTPINDNVSHCCSDSYAYR	66.7 / 97.7 / 73.7 / 63.5 / 122 / 68.4 / 134 / 149 / 68.7 / 59.1 / 73.8 / 106 / 100 / 106 / 85.8 / 72.3/ 64.4 / 55.1 / 70.9 / 61.4 / 60 / 54.9 / 59.8 / 84.6 / 75.7 / 57.2	
						CEGDMVECMDDR / RYAEFSTQLLLR / HCCSAMYAYR / FVYEYAR / FADCCSK		103 / 64 / 61 / 58 / 47
18	PREDICTED: fibrinogen alpha chain	XP_009328072.1	Pygoscelis adeliae	7.01 / 59,104.70	7 / 17.3	MSSSDGSDCSYLQGAGSVGR / TVVTSASSSFNK / LFPELNPFFTPDSSSTAGR / LLAENQNNYK / GDSLYHIAESR / NWPICADDDWGTK / SFDYQVDK	67.2 / 87 / 67.6 / 66 / 57.2 / 72.8 / 59.1	
	PREDICTED: multiple inositol polyphosphate phosphatase 1	XP_005530788.1	Pseudopodoces humilis	6.05 / 50,597.88	1 / 2	CLQSGAAFR	65.4	
	PREDICTED: beta-2-glycoprotein 1	XP_009322046.1	Pygoscelis adeliae	8.53 / 38,637.59	2 / 5.7	AVVLYNGEK / WPLNTLLCLPK	55.7 / 65	
19	PREDICTED: Gelsolin	XP_009320302.1	Pygoscelis adeliae	5.93 / 85,929.79	17/ 33.4	QGQIIYTWQGADSTR / SGAQELLK / VPFDAATLHTSK / VYVSEEGSER / AGGVASGFR / HVVPNEVTVQR / TASEFIDK / DEIATSAFLAVQLDEELGGSPVQK / HTQVQVLPESGETPLFK / TPSAAYLWVGQGASNAEK /	91.2 / 75.6 / 98.3 / 60.3 / 62.2 / 72.5 / 62 / 97.7 / 84.1 / 135 / 119 / 117 / 66.4 / 128 / 64.5 / 71.7 / 50.3	

						AVELDPTASQLNSNDAFVLK / GAAAIFTVQMDDYLQGK / VPVDPSTYGQFYGGDSYIVLYNYQHAGK / NLYGDFFTGDSYLVLNTIK / EEMLQVLGPKPSLPPGVSDETR / IEKVPFDAATLHTSK / TPVTVVK		
20	PREDICTED: prothrombin	XP_009327994.1	Pygoscelis adeliae	5.84 / 69,290.71	10 / 22.3	ELLESYMGGR / GDACEGDSGGPFVMK / IALLDNIIIHPR / TTVEFTPR / WYQVGIVSWGEGCDR / NPDNNSEGPWCYTR / SGIECQMWTSK / VMLSAPVEPCEQEK / VTDNMFCAGYSPEDSQR / VVYGDDAEVGSSPWQVMLYK	63.1 / 98.9 / 54.3 / 71.6 / 100 / 67.4 / 63.5 / 65.2 / 68.1 / 95.6	
	PREDICTED: kininogen-1	XP_009320013.1	Pygoscelis adeliae	6.41 / 68,647.09	9 / 21.2	DQFQDLTPECK / LPVEDTVNPDTCSGCPETIPK / LSEDCEATSDSAPLPCEAR / SFAVLDQPNK / TAGPDTQFYVK / AAGNQFALYVVMEAK / GGEIEAATVQVVAGQNYR / YNSESDDDFYYK / IQETTCATEENK	69.8 / 83.3 / 106 / 81.3 / 72.4 / 88.9 / 104 / 85.6 / 72.9	
21	PREDICTED: soluble scavenger receptor cysteine-rich domain-containing protein SSC5D-like	XP_009328517.1	Pygoscelis adeliae	4.67 / 50,945.95	30 / 48.1	DQIWLSDMK / DVWGTICDDQWDLR / DVWGTVCDDQWDLR / GPDMIWLDDVK / QLGCGDAVLAPIGAK / QLGCGTAVSAPR / VEVLHENR / QVGCGSPLSALGGAR / YGEGKDQIWLSDMK / WGSVCDDHWDMK / VEVLHEEQWGSVCHDNWDLNDAQVVCK	72.5 / 84.3 / 94.9 / 87 / 87.3 / 84.9 / 65.6 / 98.8 / 76.9 / 54.2/ 68.7	
						GCGSPLSALGGAR / DLNDAQVVCK / GPDMLWLDDV / GTLCDDKWELR / NASAVCSDSGLTV / SLTECEAK / QLGCSTAVSAP / VEVLHENR / HNCYH / QLGCSAAVSA / CDDQWD / AKVVCK / GELWLSD / VGGPNR / ASLTECEA / DADVVCAVNK / ECQAR / GTEVPE / VSTSVR		84 / 74 / 79 / 69 / 82 / 57 / 51 / 60 / 46 / 51 / 48 / 44 / 44 / 43 / 56 / 74 / 34 / 44 / 41
22	PREDICTED: inter-alpha-trypsin inhibitor heavy chain H2	XP_009326966.1	Pygoscelis adeliae	6.07 / 82,701 .39	9 / 17.1	AADQFSLIDFNHNVR / DNLVSATPAQVEDAK / GCCPGTLGLTPNAPNNNK / IFGNQETSAQMK / LANTMIQAK / SPPVSSLSINR / IIEPQGLR / SNALDMENFK / VDTENLQHLESIVTATAANAELVMETLR	55.2 / 92.9 / 74.8 / 92.4 / 69.1 / 77.8 / 55.5 / 57.4 / 50.8	

23	PREDICTED: alpha-2-macroglobulin	XP_009328552.1	Pygoscelis adeliae	6.04 / 165,228.83	18/ 16.5	ANAFCTSADTGFGLSPTVSLR / LVDGSGAPIANEIVR / SETLSCGSSTEVR / SGGDFQQDFQVDPTNR / TGQLSEEVK / FYATFSTPEELTETVR / HTDGSYSTFGPR / ASPNSLCAVR / ETTQNSVLCVK / MLSGFIPVK / SLIDDVVSEK / FSLLLPSNVVQDSGR / TVAWLVTPK / AVCDEFSGQTDNYGCISEMVK / VYDYYETDEFATQEYSAPCTAVK / KFEHADTACYGEEAK / VITILDEK / AIGYLVSGYQR	114 / 96.6 / 104 / 77.7 / 76.2 / 75.8 / 56.6 / 68.8 / 60.5 / 55.5 / 59.7 / 61.4 / 62.6 / 55.6 / 76.2 / 62.8 / 72.3 / 60.9
		XP_009278797.1	Aptenodytes forsteri	5.85 / 164,837.59	11 / 10.9	ANAFCTSADTGFGLSPTVSLR / ASPNSLCAVR / ETTQNSVLCVK / IKEEETGVELIGTSFSEITTTISK / LVDGSGAPIANEIVR / SETLSCGSSTEVR / SGGDFQQDFQVDPTNR / TGQLSEEVK / FSLLLPSNVVQDSGR / HTDGSYSTFGPR / SNSQSPVTFITVTVK	123 / 60.9 / 56 / 63.4 / 85.9 / 116 / 63 / 60.6 / 55.2 / 57.3 / 57.3
24 & 25	PREDICTED: complement component C7	XP_009322654.1	Pygoscelis adeliae	6.38 / 93,085.12	6 / 8.8	CFSGQCISR / CGSNLQWQVGDR / QAIEEYMAENDPCK / YSLTNAENCK / ELANLPVVYEYNIYR / LSESVLAYTFQVK /	62 / 85.3 / 84.7 / 59.8 / 68.1 / 79.4
26	PREDICTED: LOW QUALITY PROTEIN Complement C3	XP_009322238.1	Pygoscelis adeliae	6.62 / 187,591.23	1 / 1	IEGDHNAHVGLVAVDK	58.4
	Complement factor H, partial	KFW68593.1	Pygoscelis adeliae	7.47 / 126,405.91	1 / Because sequence is “partial”. its coverage was not computed	LSEYMENYK	58.1
27	PREDICTED: plasminogen	XP_009323838.1	Pygoscelis adeliae	7.82 / 90,611.88	11 / 16.3	DQQCLTLAENTK / GTASFTLTGK / NPDGEPQPWCFTTSPTK / TEGAWLLNPK / VDIALIK / TADQFPNADLK / NPDGDVNGPWCYTTDPR / YPNAGLEENYCR / CTTPPPVPAPGR / CQAWSSMSPHR / GPWCYTTDPATR	105 / 73 / 64.1 / 56.1 / 51.3 / 65.6 / 64.1 / 81.6 / 57.5 / 64.7 / 66.5
28	PREDICTED: fibrinogen alpha chain	XP_009328072.1	Pygoscelis adeliae	7.01 / 59,104.70	11 / 27	MSSSDGSDCSYLQGAGSVGR / TVVTSASSSFNK / LFPELNPFFTPDSSSTAGR / ALQSSIQEQVVEMK / LLAENQNNYK / GDSLYHIAESR / MQGLIDETDQDYSHR /	150 / 90.4 / 83.5 / 115 / 71.2 / 75.7 / 80.6 / 60.5 / 55 / 59.2 / 60

						NWPICADDDWGTK / FVTGPDGPREEVVEK / SFDYQVDK / QMQVVLER		
	PREDICTED: pyruvate kinase PKM	XP_009318184.1	Pygoscelis adeliae	7.94 / 60,260.51	1 / 2.9	FGVEQNVDMVFASFIR	60.7	
29	PREDICTED: serotransferrin	XP_009321153.1	Pygoscelis adeliae	7.61 / 77,423.99	63 / 56.5	AISNNEADAISLDSGQVFEAGLAPYK / ANIMAYR / DDSKVDDIWSFLSK / DHMQHESVALNCLQK / DSEGSAISYYTVAVVK / KGTDFTINNLQGK / WCTISSAEENK / AGEPASYFAVAVVK / DGKGDVAFVK / ESGSIEQAVANFFSASCVPGATTEQK / GTDFTINNLQGK / SDSVITWNNLQGK / VDDIWSFLSK / FGVGTTSTFQLFGPPGK / MDEFELLCTNGQR / GDVAFIK / KSDSVITWNNLQGK / GDVAFVK / HTTVQENAPEEKDEYELLCLDGSR / SKCDLWSVVSNGEVECTVADNTEDCIIK / TAPYSGYSGAFHCLK / HSIVEENTDGK / SAGWNIPIGTLLQR / VCHWATVPAHAVVAR / WCTISSAEENKCNSLR	161 / 56.3 / 84.1 / 74.6 / 119 / 101 / 79 / 109 / 63.5 / 93.7 / 82.7 / 86.7 / 77.8 / 106 / 87.3 / 54.2 / 118 / 51.3 / 80.1 / 64.5 / 71.3 / 74.6 / 59.4 / 91.2 / 91.7	
						ADALSLDSG / ADALSLDSGKVFEAAVAPYK / ANLMAYR / APGSPPNSR / APYSGYGSAFH / ATYLDCLK / CAVGK / DEFELLCTNGQR / DGKGDVAFVK / DLLFK / DSALKLK / EDMTQWC / EFELLCTNG / EKDEYELLCLDGSR / EPASYFAVAVVK / FFAVAVAR / FTLNNLQGK / GSALSYYTVAVVK / HSLVEEETNGK / KGTDFTLNNLQGK / KVDDLWSFLSK / LCQLC / LCELCK / LQPLAAEVY / NFEEYFSEGCAPGSP / NLPLGTLLKR / NPSDLLQVCTFL / AVANFFSASCV / SALQSL / SYYTVAVV / TSCHTGLGR / TTSTFELFGP / VALNCLQK / WCTLSSAEENK / WNNLQGK / WNVLSN / YFSEGCAPG / YYGYTGAFR		48 / 112 / 54 / 64 / 66 / 58 / 36 / 90 / 71 / 34 / 34 / 36 / 66 / 102 / 87 / 37 / 90 / 63 / 63 / 91 / 81 / 38 / 38 /52 / 110 / 54 / 86 / 88 / 36 / 60 / 64 / 93 / 56 / 84 / 55 / 34 / 35 / 72
30	PREDICTED: angiotensinogen	XP_009327462.1	Pygoscelis adeliae	5.10 / 50,279.95	12 / 31.4	AVDFTNPGEAAK / ISDANLTVGK / LASGGTDQPEDPAAQK / MIESLIESR / TDASGIFSVVEVPISK / IEGSSDLQELLADMELPALLGK /	80.6 / 88.7 / 74.8 / 69.7 / 108 / 91.5 / 56.2	

					STCEELESLVQEGK		
					DLDPSTNLLMVVDVR / EPKEFWVDSN / AVDFTNPGEAAK / QLNAFVEAK / VPFLSVTGTFK		93 / 72 / 86 / 64 / 65
PREDICTED: fetuin-B	XP_009320015.1	Pygoscelis adeliae	5.01 / 45,162.38	2 / 4.9	AIIYINQAR / YLEAAVQSLAK	66.4 / 95.8	

Table S1 summarizes annotations of all the identified proteins, which were extracted from the Scaffold software (Proteome Software, v3.6.5), computed from www.expasy.org/tools/pi-tool, or extracted from MS-Blast searches. The theoretical isoelectric points (pI) and molecular weights (MW) of identified *Pygoscelis adeliae* proteins were consistent with those observed on 2-D gels.

The MS/MS data were interpreted using a local Mascot™ server (Mascot version 2.5.1, Matrix Science, London, UK). Spectra were searched with a tolerance of 0.5 Da on mass measurements, both in MS and MS/MS modes. A maximum of one trypsin missed cleavage per peptide was allowed, and carbamidomethylation of cysteine, oxidation of methionine and acetylation of protein N-termini were specified as variable modifications. The Scaffold software (v.3.6.5, Proteome software Inc., Portland, OR, USA) was used in order to validate identifications using the following criteria. Hence, spectra were searched against a target-decoy version of the NCBInr database restricted to *Aves* (Taxonomy ID: 8782) downloaded in July 2015 (3270982 target+decoy entries). Protein identifications were validated when peptides with high quality MS/MS spectra were detected (in the case of more than 2 peptides-based identification, MS/MS ion scores higher than 39.7; in the case of single peptide-based identification, MS/MS ion score higher than 57).

In addition to classical Mascot searches, complete or partial amino acid sequences were determined on the non-assigned MS/MS spectra through automatic *de novo* sequencing, which was performed using the PEAKS Studio software (v4.5; Bioinformatics Solutions, Waterloo, Canada). All deduced sequence tags were submitted to the MS-BLAST program to perform cross-species protein identifications on the basis of sequence similarity. Identifications were validated for best hits according to threshold scores, which were as follows: 102 for the summed score of 2 HSPs, 136 for the summed score of 3 HSPs, and 200 for the summed score of 4 or more HSPs.

Table S2. Annotations relative to single peptide-based protein identifications, with corresponding annotated mass spectra

Spot N°	Protein	NCBI Accession.version	pI / MW (Da)	Peptide sequence	Mascot MS/MS ion score	Modifications	Observed m/z	Actual peptide mass (AMU)	Charge	Error on peptide mass (PPM)
9	PREDICTED: LOW QUALITY PROTEIN: immunoglobulin lambda-like polypeptide 1	XP_009329120.1	6.41 / 24741.85	QSNNKYMASSYLTLSASDWK	97.6	Oxidation	1155.58	2309.14	2	34
	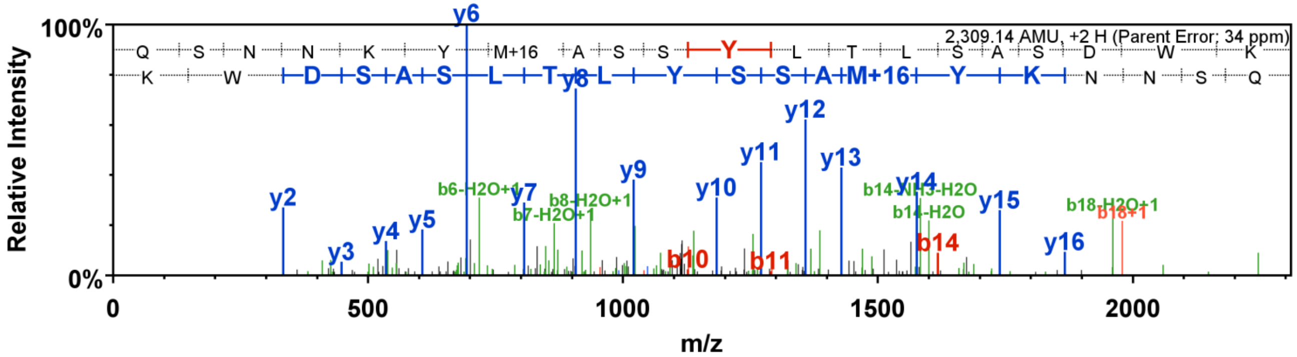									
12	PREDICTED: LOW QUALITY PROTEIN: immunoglobulin lambda-like polypeptide 1	XP_009329120.1	6.41 / 24741.85	QSNNKYMASSYLTLSASDWK	108	Oxidation	1155.58	2309.14	2	34
	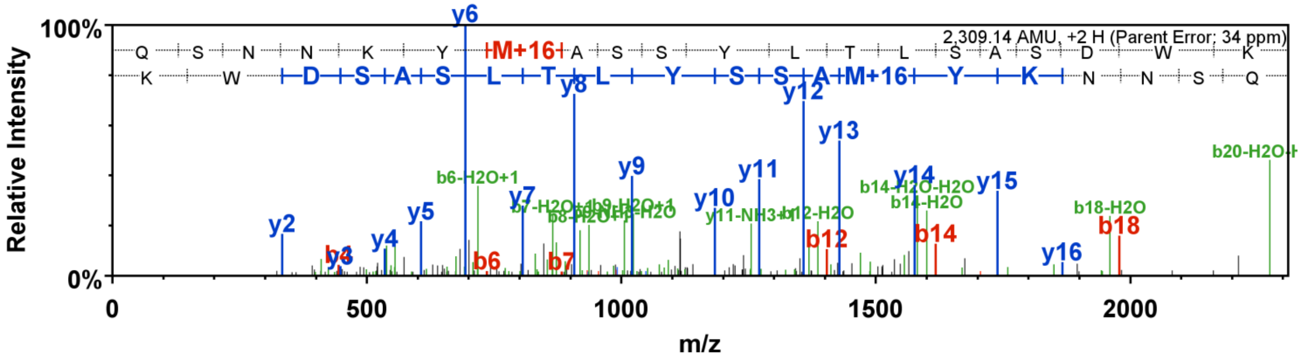									

Ig lambda chain V-1 region, partial	KFW61302.1	6.19 / 6395.03	VPGSAPVTVIYANDK	114	None	765.89	1,529.76	2	-31

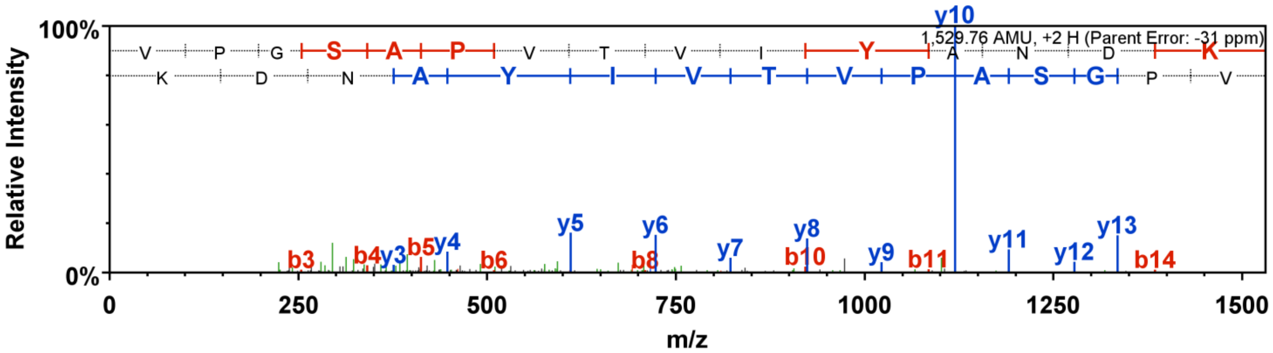

12

Ig lambda-1 chain C regions, partial	KFQ94409.1	6.91 / 11720.02	ATLVCLLGDFYPR	97.5	Carbamido-methylation	762.88	1,523.75	2	-18

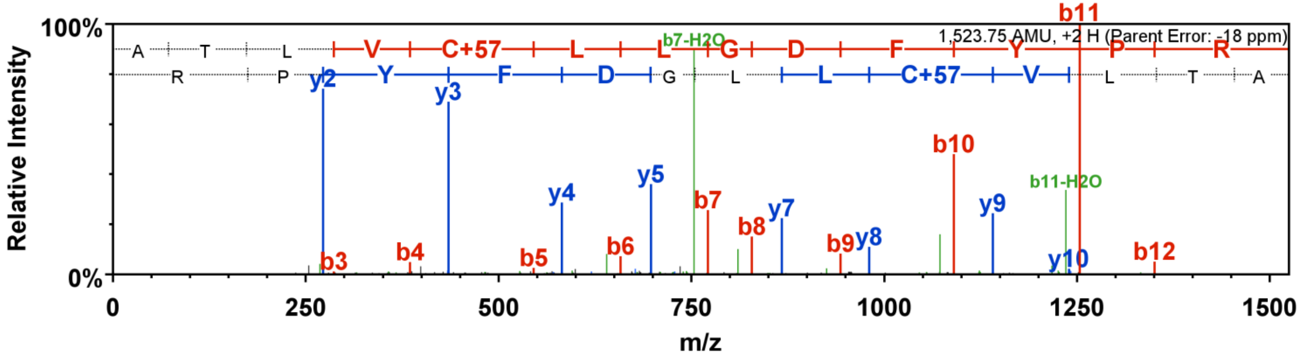

	lactate dehydrogenase A	XP_011574069.1	8.19 / 36602.51	VIGSGCNLDSAR	68.7	Carbamido-methylation	624.82	1247.62	2	23
13	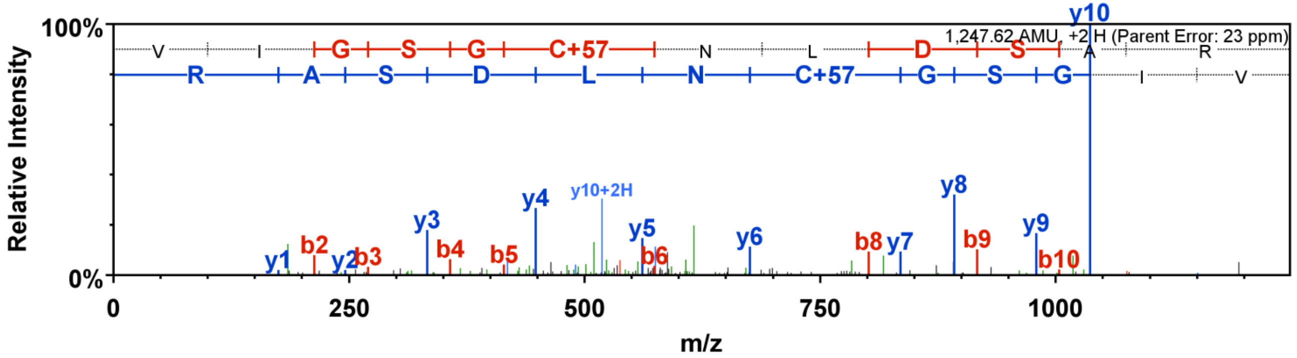									

	Ig gamma chain C region, partial	EMC81142.1	4.72 / 10826.22	GFYPEDVSVEWQK	76.7	None	792.40	1,582.79	2	36
15	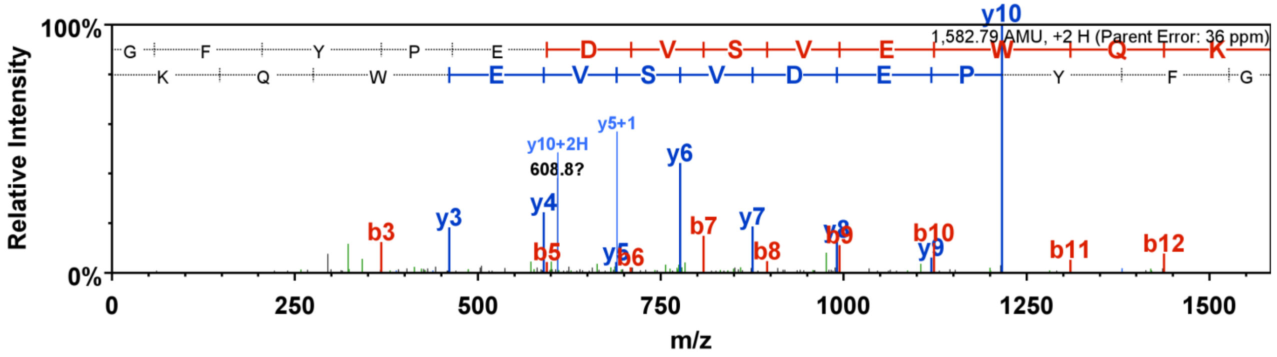									

	Ig heavy chain V-III region VH26, partial	KFQ92087.1	5.10 / 6711.43	STVTLQMNSLK	59.5	Oxidation	619.32	1,236.62	2	-17

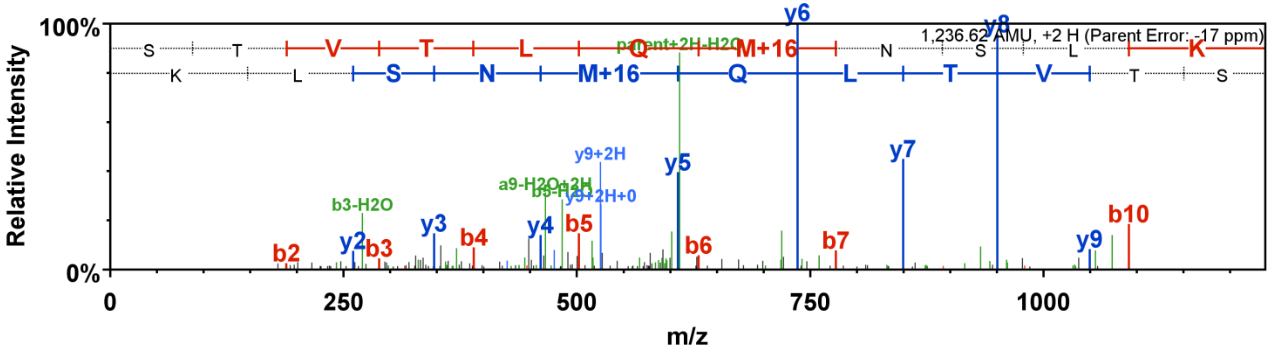

15	Ig heavy chain V-III region VH26, partial	KFP43328.1	8.11 / 6122.75	ADDTATYYCAK	72.4	Carbamido-methylation	631.82	1,261.63	2	78

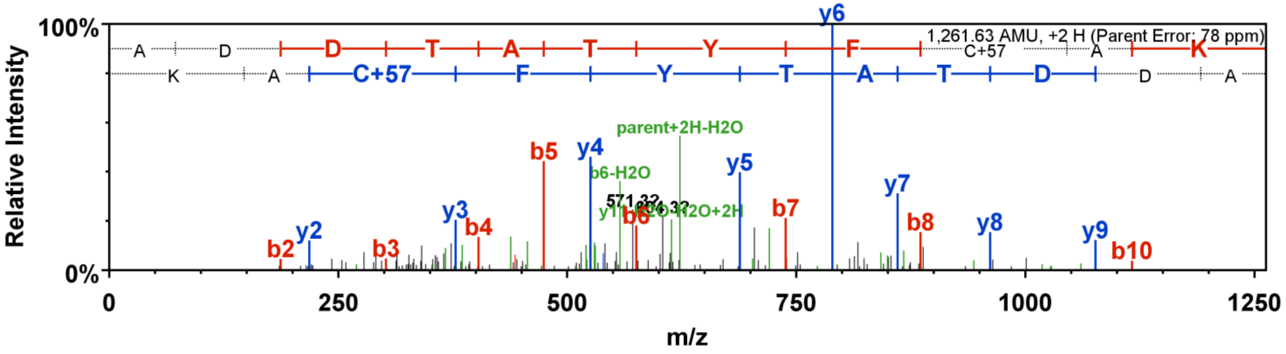

18	PREDICTED: multiple inositol polyphosphate phosphatase 1	XP_005530788.1	6.05 / 50597.88	CLQSGAAFR	65.4	Carbamido-methylation	505.26	1,008.50	2	22

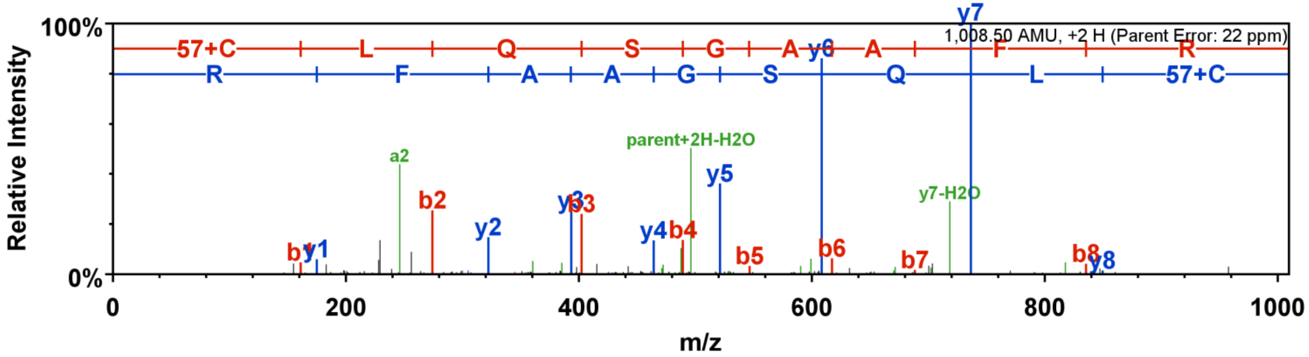

26	PREDICTED: LOW QUALITY PROTEIN Complement C3	XP_009322238.1	6.62 / 187591.23	IEGDHNAHVGLVAVDK	58.4	None	558.62	1,672.82	3	-18

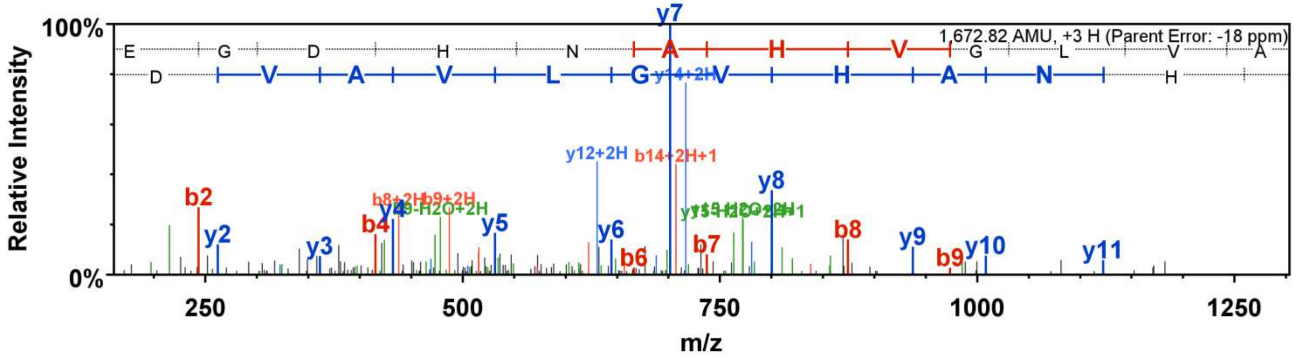

Complement factor H, partial	KFW68593.1	7.47 / 126405.91	LSEYMENYK	58.1	Oxidation	596.81	1,191.60	2	74

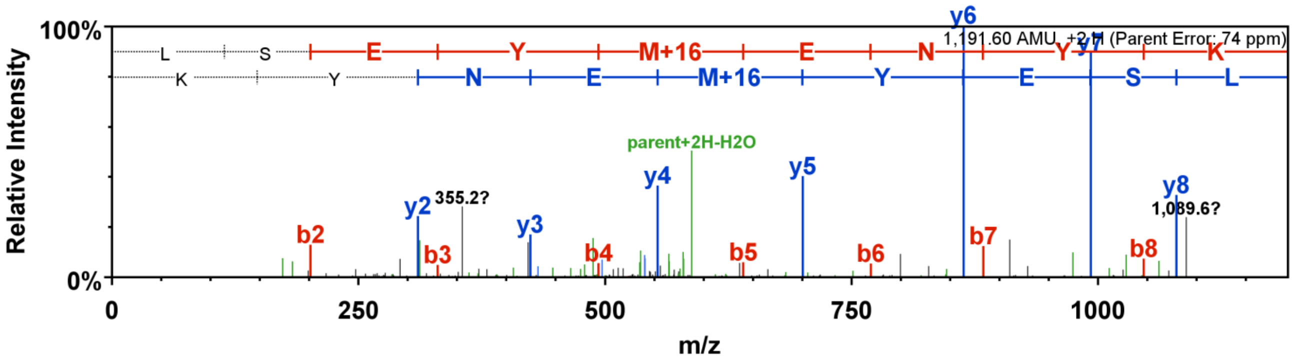

Pyruvate kinase muscle isozyme	XP_009318184.1	7.94 / 60260.51	FGVEQNVDMVFASFIR	60.7	None	930.1	1,858.19	2	150

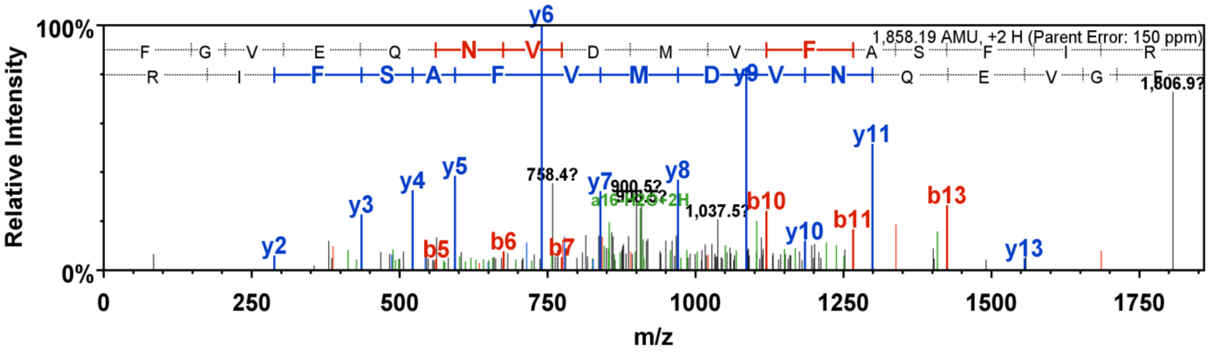

All information was extracted from the Scaffold software (Proteome Software, version 3.6.5), or computed using www.expasy.org/tools/pi-tool.
